# *In vitro* and *in vivo* toxicity and antibacterial efficacy of melittin against clinical extensively drug-resistant bacteria

**DOI:** 10.1186/s40360-021-00503-z

**Published:** 2021-07-14

**Authors:** Parvin Askari, Mohammad Hasan Namaei, Kiarash Ghazvini, Mehran Hosseini

**Affiliations:** 1 Department of Microbiology and Virology, Faculty of Medicine, Mashhad University of Medical Sciences, Mashhad, Iran; 2 Infectious Diseases Research Center, Birjand University of Medical Sciences, Mashhad, Iran; 3 Antimicrobial Resistance Research Center, Mashhad University of Medical Sciences, Mashhad, Iran; 4 Department of Anatomical Sciences, Faculty of Medicine, Birjand University of Medical Sciences, Birjand, Iran

**Keywords:** Antimicrobial Drug Resistance, Antimicrobial Peptides, Melitten, Toxic Potential, Animal model, Sepsis

## Abstract

**Background:**

Melittin is one of the most studied antimicrobial peptides, and several *in vitro* experiments have demonstrated its antibacterial efficacy. However, there is evidence showing melittin has non-promising effects such as cytotoxicity and hemolysis. Therefore, concerns about unwanted collateral toxicity of melittin lie ahead in the path toward its clinical development. With these considerations, the present study aimed to fill the gap between *in vitro* and *in vivo* studies.

**Methods:**

In the first step, *in vitro* toxicity profile of melittin was assessed using cytotoxicity and hemolysis tests. Next, a maximum intraperitoneal (i.p.) sub-lethal dose was determined using BALB/c mice. Besides toxicity, antimicrobial efficacy of melittin against extensively drug-resistant (XDR) *Acinetobacter baumannii*, methicillin-resistant *Staphylococcus aureus* (MRSA), and KPC-producing *Klebsiella pneumonia* (KPC-KP) pathogens were tested using both *in vitro* and *in vivo* methods.

**Results:**

Melittin showed extensive hemolysis (HD_50_ = 0.44 µg/mL), and cytotoxicity (IC_50_ = 6.45 µg/mL) activities with i.p. LD_50_ value of 4.98 mg/kg in BALB/c mice. *In vitro* antimicrobial evaluation showed melittin MIC range from 8 to 32 µg/mL for the studied pathogens. Treatment of infected mice with repeated sub-lethal doses of melittin (2.4 mg/kg) displayed no beneficial effect on their survival and peritoneal bacterial loads.

**Conclusions:**

These results indicate that melittin at its safe dose could not exhibit antimicrobial activity, which hinders its application in clinical practice.

## Background

Antibiotics have saved millions of lives around the world since the early 20th century. However, in the last decade, the emergence of drug-resistant pathogens has represented a serious threat to public health [1]. Antibiotic resistance may occur naturally, but misuse and overuse of antibiotics, lack of standard treatment guidelines, and misuse in animal husbandry are responsible for accelerating the process [2]. According to estimates of the U.S. centre for disease control and prevention, antibiotic resistance causes at least 2.8 million infections and 35,900 deaths per year in the United States alone. As antibiotics become less effective, management of a growing list of infections is becoming challenging and occasionally impossible. Therefore, antibiotic resistance is considered the most significant public health problem of our time [3]. Currently, high rates of antibiotic resistance for common bacterial infections such as urinary tract infections, sepsis, and some forms of diarrhea are reporting worldwide [4]. These accumulating reports indicate the fact that we are running out of effective antibiotics. Subsequently, it is crucial to develop new effective treatments against highly resistant bacteria. There are different patterns of resistance among bacteria, including multiple drug resistance (MDR), extensively drug-resistant (XDR), and pandrug-resistant (PDR) [5].

Currently, three promising alternative approaches to fight these resistance patterns are antimicrobial peptides (AMPs), nanoparticles, and the design of novel combinatorial therapies [6].

AMPs are ancient components of the innate immune defense system in all life classes and recently have emerged as promising candidates for drug development. To date, more than 3000 AMPs have been characterized and evaluated, but a limited number of them approved by the U.S. Food and Drug Administration [7]. Melittin, the main component of the bee venom, is one of the most studied AMPs with a broad spectrum of biological activities. It contains 26 amino polypeptides with a molecular mass of 2840 Da. The N terminal of this polypeptide has 4^+^ charges, whereas its C terminal has 2^+^ charges at physiological pH. As a result, in normal physiological conditions, melittin forms a monomeric alpha-helix when bound to the cell membrane’s lipid bilayer, which helps it penetrate the cell membrane and molecularly act on cellular sub-structures [8]. A series of recent studies indicated that melittin has a wide range of bactericidal activity against susceptible and resistant bacteria [9–12]. Despite a tremendous amount of works done in beneficial activities of melittin, some studies have highlighted the potential specific toxic effects of this peptide [7, 13, 14]. Many experimental studies have only focused on investigating the benefits of melittin, and the *in vivo* data on its effectiveness and safety in systemic administration is quite limited.

Hence, this study was firstly designed to determine *in vitro* and *in vivo* toxicity of melittin. In the next step, the efficacy (*in vitro* and *in vivo*) of the safe dose of melittin was investigated against the three most common nosocomial bacterial pathogens, including XDR *Acinetobacter baumannii*, methicillin-resistant *Staphylococcus aureus* (MRSA), and KPC-producing *Klebsiella pneumonia* (KPC-KP).

## Methods

### Peptide synthesis

The synthetic melittin peptide was obtained from a yeast expression system as previously described [15]. Briefly, the sequence of melittin was cloned in the pPIC9 vector and then transformed into the *Pichia pastoris* GS115. Finally, a product yield of 105 µg melittin/L was achieved. During the peptide synthesis and purification, a standard melittin peptide obtained from Sigma-Aldrich (St. Louis, MO, USA) was used.

### Cytotoxicity assay

The Human primary fibroblast cell line (C654, Pasteur Institute of Iran, Iran) was grown in Dulbecco’s modified Eagle’s medium (DMEM) supplemented with 10 % fetal bovine serum (FBS) (Biosera Co. France). Once established, fibroblast cultures were trypsinized (Gibco, Canada) and transferred into a polystyrene 96-well plate with 3 × 10^3^ cells per well in 200-µL medium and incubated at 37 °C in 5 % CO_2_ for 12-h or when they reached 80 % confluence. Afterward, the medium was replaced with PBS (phosphate-buffered saline) overnight for cell starving, and then cells were exposed to melittin at the two-fold concentration (0.625-10 µg/mL) for 24-h at 37 °C in 5 % CO2. The control wells were maintained with PBS. The 3-(4, 5-dimethylthiazol-2-yl)-2, 5-diphenyltetrazolium bromide (MTT) dye was used for cytotoxicity evaluation. The cells were rinsed with PBS and incubated with 0.5 mg/mL MTT diluted in complete DMEM for 4-h. Then, supernatants were removed, and 150 mL of dimethyl sulfoxide was added, and the plate was incubated for 10 min. The absorbance was read at 570 nm using a 96-well ELISA plate reader (BioTek, Vermont, USA). Five replicates for all concentrations were performed. The percentage of cell viability was calculated as follows: percentage of cell viability = [(A treatment – A blank)/(A control – A blank)] × 100 (where, A = absorbance)[16].

### Hemolysis assay

The hemolysis assay was carried out as previously reported by Evans et al. [17]. Briefly, heparinized blood samples were collected from healthy volunteers. Red blood cells (RBCs) were extracted from the whole human blood sample by removing white blood cells and platelets through centrifugation (500 rpm, 5 min). The remained RBCs were washed in PBS. The melittin was dissolved in PBS and added to the suspension of RBCs (2 % final in v/v in PBS) at a concentration range from 0.061 to 15.6 µg/mL and incubated at 37 °C for 1-h. The samples were centrifuged (500 rpm, 5 min), supernatants collected, and 100 µL of each sample’s supernatant was transferred into a 96-well plate. The hemoglobin release was evaluated by measuring the absorbance (A-sample) of the samples at 540 nm. For negative and positive controls, PBS (A-blank) and Triton X-100 (A-Triton) (0.2 % v/v) were used, respectively. The percentage of hemolysis was calculated according to the equation.

Percentage of hemolysis = [(A-sample – A-blank)/ (A-Triton – A-blank)] × 100.

### In vivo toxicity assessment

#### Animals

Male adult BALB/c mice (8-week old) weighing 20–25 g were used in the present study. All animals were housed in polypropylene cages in a temperature-controlled room (24 ± 2ºC) with 30–35 % relative humidity and a 12-h light/dark cycle. Mice were given free access to water and standard chow during the study period. All procedures involving animals were in accordance with the national guides in care and use of Laboratory Animals in Scientific Affairs provided by the Iranian Ministry of Health and Medical Education (2020). The guideline complies with the ARRIVE guidelines [18]. Moreover, all the study protocols, including the animal experiments, were approved by the Mashhad University of Medical Sciences Ethics Committee (permit code: IR.MUMS.MEDICAL.REC.1399.151).

In the present study, two main types of experiments were used to assess melittin toxicity in mice:

(I) Acute toxicity assessment upon single-dose administration of melittin.
(II) Sub-acute toxicity assessment upon multi-dose administration of melittin with different intervals.

#### Acute toxicity experiment

A two-fold concentration gradient test was carried out to determine the *in vivo* LD_50_ (median lethal dose) value for melittin. Accordingly, four different doses (1.2 mg/kg, 2.4 mg/kg, 4.8 mg/kg, and 9.6 mg/kg) were tested. For each concentration, five mice were injected intraperitoneally (i.p.) once and monitored for five days [19].

#### Sub-acute toxicity

Evaluation of sub-acute toxicity was conducted with two-time intervals (12-h vs. 8-h) to assess the probable cumulative toxic effect. Firstly, melittin at the dose of 2.4 mg/kg (sub-lethal dose determined in acute assay) was injected (i.p.) into mice (*n* = 10 each), every 12-h for five consecutive days (totally 11 injections). During the study period, animals were monitored daily for mortality, changes in their fur, eyes, mucous membranes, and behavioral signs (salivation, tremors, convulsions, diarrhea, and lethargy). In order to determine the safe dosing interval, additional multi-dose treatments were performed by injection of 2.4 mg/kg of melittin into other mice (*n* = 10 each), every 8-h for five consecutive days (totally 16 injections) [19]. Finally, animals were anesthetized with ketamine-xylazine (100:10 mg/kg i.p.), and blood samples were collected via cardiac puncture. Blood samples of half of the mice from each group were collected into tubes containing no anticoagulants. Samples allowed to clot, centrifuged (3000 g for 15 min), and sera were obtained for blood chemistry. Biochemical evaluation of creatinine, urea, aspartate transaminase (AST), and alanine transaminase (ALT) were performed. The blood samples of another half of the mice from each group were collected in ethylene diamine tetra-acetic acid (EDTA) tubes for hematological evaluation. Immediately after the blood collection, liver and kidney (left kidney) were dissected out, weighed, and fixed in 4 % paraformaldehyde solution for histological evaluation.

### In vitro and in vivo antibacterial efficacies

#### Bacterial strains and reagents

In the present study, different strains of the three most common pathogens responsible for nosocomial infections were used. The pathogens were including XDR *A. baumannii*, MRSA, and KPC-KP. DNA sequencing analysis was performed for confirming *A. baumannii* using 16 S rRNA specific primer and PCR method. *S. aureus* strains were also confirmed using standard culture and biochemical tests. Multiplex PCR was used to detect XDR (*bla*_IMP_, *bla*_VIM_, and *bla*_NDM_ genes) and MRSA (*mceA* gene) strains as previously reported [20, 21]. In addition, a clinical isolate of KPC-producing *Klebsiella pneumonia* (KPC-KP-C11) with positive *KPC*, *NDM*, and *OXA-48* genes was chosen from previous work [22]. All media, including Mueller-Hinton broth (MHB), Mueller-Hinton agar (MHA), Brain heart infusion Broth (BHIB), Blood-agar (BA), and Trypticase soy broth (TSB), were purchased from Merck (Germany). Antibiotics including colistin sulfate (CAS No. 1264-72-8) and vancomycin hydrochloride (CAS No. 1404-93-9) were purchased from Sigma (USA).

#### *In vitro* antibacterial evaluation

The minimum inhibitory concentrations (MICs) of melittin, vancomycin and colistin were determined by the broth microdilution method proposed by the Clinical and Laboratory Standards Institute [23]. Briefly, the numbers of 1.5 × 10^5^ (CFU/mL) bacteria were added to each well of a polystyrene 96-well plate containing different concentrations of melittin (2-100 µg/mL), vancomycin (0.125-128 µg/mL), and colistin (0.25–128 µg/mL) and incubated at 37° for 16–20 h. All experiments were performed in duplicate. The MIC was defined as the lowest concentration of the agent (peptide or antibiotic) to produce complete inhibition of visible growth.

In the next step, to determine the minimum bactericidal concentrations (MBCs) of melittin, vancomycin, and colistin, two 10µL samples from each well containing no visible growth were sub-cultured on MHA medium and incubated at 37° for 12–18. The MBC was defined as the concentration that killed all the tested bacteria (99.9 % killing) [9].

In order to observe the dynamic picture of the bactericidal activity of melittin, the time-killing curve (TKC) assay was carried out as previously described [9]. Briefly, a strain from each of the studied organisms was cultured in MHB medium at 37 °C for 24-h. Afterward, 10^7^ CFU/mL of each strain was added to every well of a polystyrene 96-well plate in the presence of melittin at 1–4× MICs. Eventually, 10µL from each well was sampled at different time points (0, 0.5. 2, 3, 5, and 24-h) after incubation and sub-cultured on MHA medium at 37 °C for 24-h. Experiments were performed in duplicate. TKCs were constructed by plotting mean colony count (log _10_ CFU/mL) [19].

#### *In vivo* antibacterial evaluation

After *in vivo* toxicity assessment of melittin and determination of sub-lethal dose, this phase of the study was performed. The *in vivo* antibacterial efficacy of melittin was evaluated in three mouse models of intraperitoneal infection induced by the three most common nosocomial pathogens.

#### Mouse peritoneal model of *A. baumannii*

A mouse model of *XDR A. baumannii* sepsis was developed as previously described [24, 25]. Briefly, all mice were rendered neurotropic by i.p. injection of cyclophosphamide at 150 mg/kg (200 µL), 4 and 1 days before infection. Firstly, the minimal lethal dose of *A. baumannii* was determined by pilot experiments in which different doses of *A. baumannii* (XDR-CI66) (10^3^-10^9^ CFU/mouse) were administrated (single i.p.) to the neurotropic animals. The minimal lethal dose was considered a dose able to kill 100 % of the infected neurotropic animals (LD_100_) over 72-h. The minimal lethal dose of *A. baumannii* (XDR-CI66) was determined as 10^7^ CFU/mouse.

For survival analysis, the neurotropic mice were infected by the LD_100_ of *A. baumannii* (10^7^ CFU/mouse dissolved in 500µL PBS) and then randomly were divided into three main groups (*n* = 10 each), including untreated, melittin treated, and colistin treated. PBS, melittin (2.4 mg/kg), and colistin (1.5 mg/kg) were administrated (i.p.) to control untreated, melittin treated and colistin treated groups respectively started 1-h post-infection and repeated every 12-h up to 36-h (totally four injections). The animals were monitored for five days.

In order to estimate the peritoneal load of *A. baumannii*, mice were infected by a sub LD_100_ dose of *A. baumannii* (10^6^ CFU/mouse). Then, the infected animals were randomly divided into three groups (*n* = 15 each), including untreated (PBS), melittin treated (2.4 mg/kg), and colistin treated (1.5 mg/kg). Treatments were started 1-h post-infection and repeated every 12-h up to 36-h (totally four injections). Every 12-h post-infection, five mice from each group were euthanized, their peritoneal cavities were lavaged with 5mL sterile chilled saline, and their blood samples were also collected. Blood samples were used for bacterial culture, and lavage fluids were firstly aliquoted into 10-fold serial dilutions and then cultured on the BA medium to quantify the number of viable *A. baumannii* in the respective samples.

#### Mouse peritoneal model of *Staphylococcus aureus* (MRSA)

Like the *A. baumannii* peritoneal model, mice were immunosuppressed and then subjected to LD_100_ and a sub-lethal dose of MRSA for survival and antibacterial evaluations. The LD_100_ of MRSA was determined through pilot experiments in which different doses (10^4^-10^9^ CFU/mouse) of *S. aureus* (MRSA-CI66) were administrated. The LD_100_ was considered a dose able to kill 100 % of the infected neurotropic animals over 36-h [26]. The LD_100_ for the tested *S. aureus* was determined as10^7^ CFU/mouse.

Mice were infected by a single i.p. injection of the LD_100_ dose of MRSA (10^7^ CFU/mouse). The infected mice were randomly allocated into three main groups (*n* = 10 each) including, untreated (PBS), melittin treated (2.4 mg/kg), and vancomycin treated (200 mg/kg). The dose of vancomycin was optimized according to previous works and pilot experiments in which three doses of this drug (25, 150, and 200 mg/kg ) were tested. Vancomycin (200 mg/kg subcutaneously) was only administrated once at 0.5-h after infection [27]. The untreated and melittin treated groups were treated with PBS, and melittin (2.4 mg/kg i.p.) respectively initiated 1-h post-infection and every 12-h up to 36-h (totally four injections). Similar to *A. baumannii* sepsis, survival analysis was performed for MRSA.

In order to estimate the peritoneal load of MRSA, mice were infected by a sub LD_100_ dose of MRSA (10^6^ CFU/mouse). Then, the infected animals were randomly divided into three groups (*n* = 15 each) including, untreated (PBS), melittin treated (2.4 mg/kg), and vancomycin treated (200 mg/kg). Treatments were similar to those performed in survival assessment. Every 12-h post-infection, five mice from each group were euthanized, their blood and lavage samples were collected and used for bacterial culture and quantifying the number of viable MRSA in the respective samples.

#### Mouse peritoneal model of *Klebsiella pneumonia* (KPC-KP)

Mice were immunosuppressed as described above. Like the other studied bacteria strains, the LD_100_ of KPC-KP was determined. Briefly, different doses (10^6^-10^9^ CFU/mouse) of KPC-KP were administrated, and the minimum dose that was able to kill 100 % of the infected neurotropic animals over 72-h was considered as LD_100_ [28]. The minimal lethal dose of K pneumonia (CI1 KPC-KP) was determined as 10^8^ CFU/mouse.

The immunosuppressed mice were infected by a single i.p. injection of the LD_100_ of KPC-KP (10^8^ CFU/mouse). The infected mice were randomly allocated into three main groups (*n* = 10 each), including untreated (PBS), melittin treated (2.4 mg/kg), and colistin treated (12.5 mg/kg). The colistin dose was optimized according to previous works and pilot experiments in which three doses of this drug (2.5, 12.5, and 25 mg/kg) were tested. Treatments with PBS, melittin (2.4 mg/kg), and colistin (12.5 mg/kg) were initiated at 1-h post-infection and repeated every 12-h up to 72-h (totally seven injections). For survival analysis, the animals were monitored for five days.

In order to estimate the peritoneal load of KPC-KP, the immunosuppressed mice were infected by a sub LD_100_ dose of KPC-KP (10^7^ CFU/mouse). Then, the infected animals were randomly divided into three groups (*n* = 15 each). Grouping and treatments were similar to those performed in the survival assessment. Blood and lavage samples were collected at 24, 36, and 48-h post-infection and used for bacterial culture and quantifying the number of viable KPC-KP in the respective samples.

### Statistical analysis

Data are presented as mean ± standard deviation (SD) for each group unless otherwise specified. Differences in quantitative measurements were assessed by one-way analysis of variance followed by Dunnett T3 post-hoc multi-comparison test, Kruskal Wallis, and Student’s t-tests, when appropriate. A log-rank test was run to determine differences in the survival distribution for the different types of intervention. The number of repetitions is given in the method. Differences were considered significant when *P* < 0.05.

## Results

### *In vitro* toxicity results

**Fig. 1:**
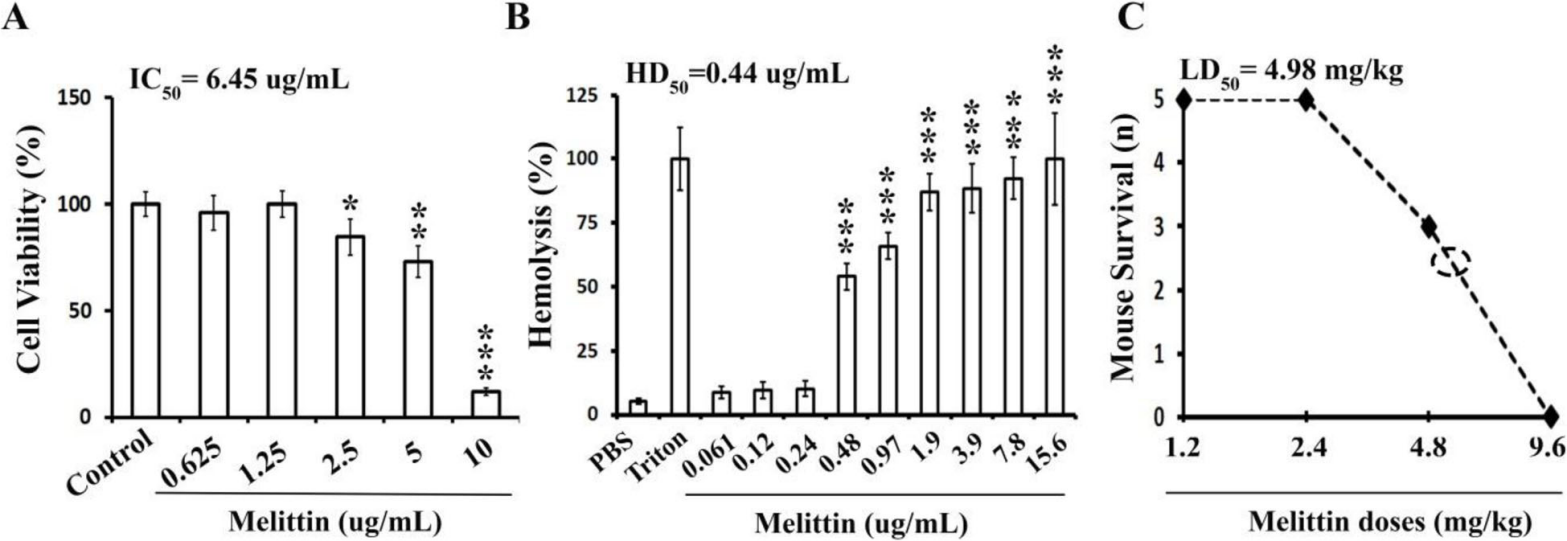
*In vitro* and *in vivo* toxicity results of melittin peptide. (**A**) Cytotoxicity of melittin peptide (0.625-10 µg/mL) on human primary fibroblast cells by the MTT assay. The half-maximal inhibitory concentration (IC_50_) of melittin to fibroblast cells was 6.45 µg/mL. (**B**) Human blood hemolysis assay for melittin peptide (0.061–15.6 µg/mL) in which Triton X-100 and phosphate buffered saline (PBS) were used as positive and negative controls, respectively. Results (cytotoxicity and hemolysis) are expressed as mean ± SD. Statistical differences in relation to the control group (100 %) are represented as * p < 0.05, ** p < 0.01 and ***p < 0.001. (**C**) *In vivo* intraperitoneal LD_50_ (median lethal dose) determination for melittin using BALB-c mice (*n* = 5)

The cytotoxicity evaluation of melittin in human fibroblast cells was performed by MTT test. The results revealed that the viability was affected by melittin at concentrations greater than or equal to 2.5 µg/mL (Fig. 1 A). The melittin IC_50_ was calculated using a linear regression equation, and it was 6.45 µg/mL.

*In vitro* hemolysis test was performed using human RBCs. Melittin exhibited a significant hemolytic activity (Fig. 1B). The melittin concentration for 50 % hemolysis (HD_50_) was 0.44 µg/mL, and at concentrations more than 1 µg/mL, its hemolytic activity was about 80–90 %.

### Acute toxicity results

Based on the acute toxicity assay results, the i.p. LD_50_ value for melittin was 4.95 mg/kg. The maximum i.p. sub-lethal dose for melittin was 2.4 mg/kg (Fig. 1 C).

### Sub-acute toxicity results

In cumulative toxicity assessment, repeated injections (11 injections in 12-h-interval or 16 injections in 8-h-interval experiments) of the sub-lethal dose of melittin (2.4 mg/kg) were performed in mice for five consecutive days. The results showed that 10 of 10 injected mice in sub-acute experiments (both studied intervals) were survived. However, the behavioral assessment showed that all mice presented clinical signs of pain immediately after the melittin injection, including back-arching, belly-pressing, twitching, and staggering. These signs were disappeared over the course of a few minutes (up to 5 min) after injection.

The biochemical evaluation revealed no significant difference in biochemical parameters (AST, ALT, urea, and creatinine) between control and melittin-treated mice (Table 1.). However, the hematological assessment showed that the WBC count of melittin-treated mice was significantly lower than the control group (*P* = 0.004).

**Table 1:** Biochemical and hematological results in sub-acute experiment (16 injections with 8-h intervals)

Groups	Urea (mg/dL)	Creatinine (mg/dL)	AST (U/L)	ALT (U/L)	RBC (10^6^/µL)	WBC (10^3^/µL)
**Control**	44.33 ± 2.25	0.55 ± 0.05	79.00 ± 26.39	58.00 ± 21.37	9.64 ± 0.86	6.5 ± 0.23
**Melittin 2.4 mg/kg**	46.83 ± 2.31	0.52 ± 0.02	67.00 ± 5.36	61.00 ± 7.64	9.20 ± 0.60	5.06 ± 0.42
**p-value (t-test)**	*0.08*	*0.22*	*0.32*	*0.75*	*0.32*	*0.004*

AST: aspartate transaminase; ALT: alanine transaminase; RBC: red blood cell; WBC: white blood cell

**Fig. 2:**
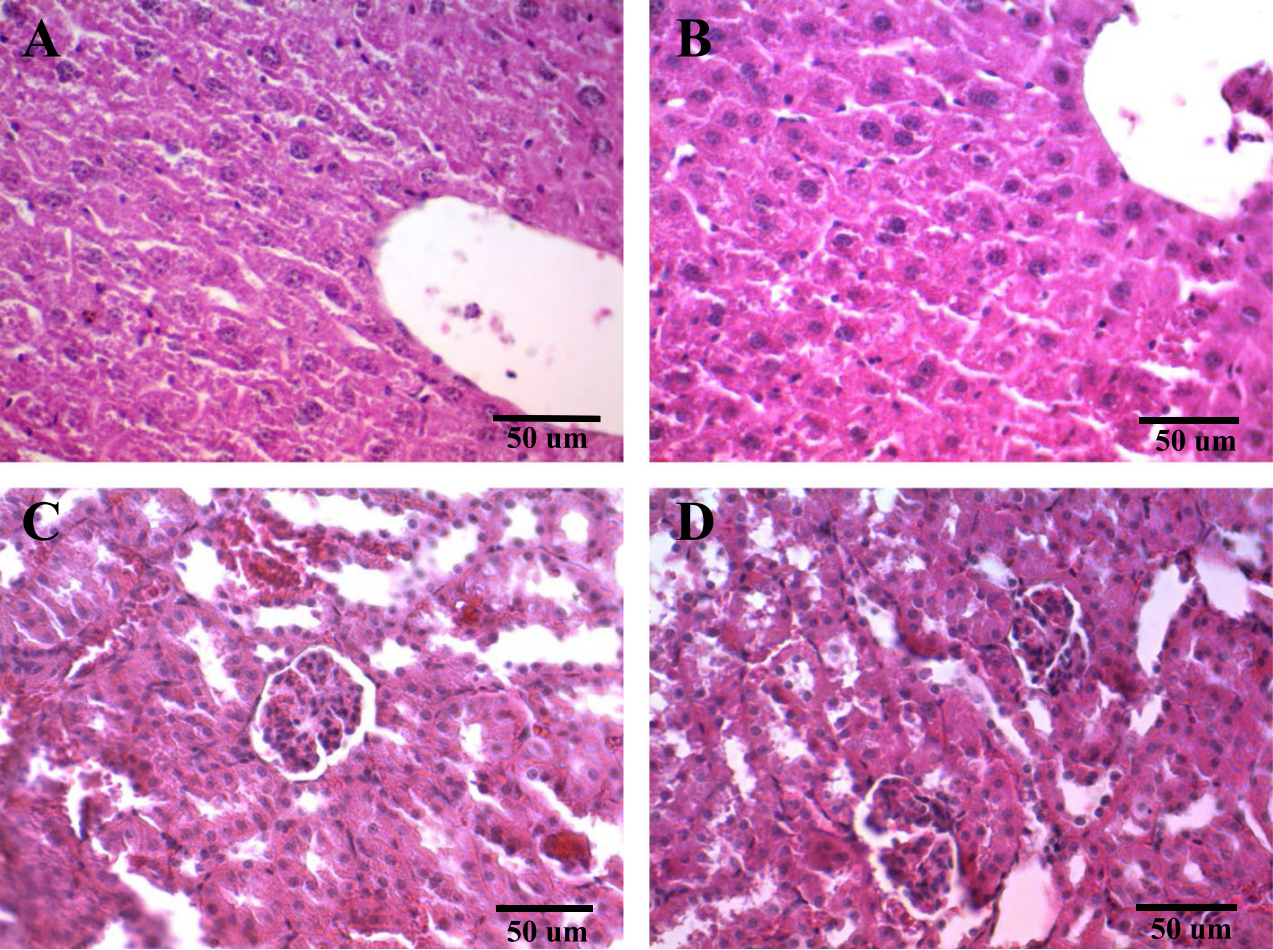
Photomicrographs of liver and kidney sections stained with hematoxylin and eosin dyes (400X, scale-bars = 50 μm). (**A**) Liver micrograph from an untreated mouse, displaying no damage. (**B**) Liver micrograph from multi-dose administration (16/8 h intervals) of melittin (2.4 mg/kg), displaying no damage. (**C**) Kidney micrographs from an untreated mouse showing no damage. (**D**) Kidney micrograph from multi-dose administration (16/8 h intervals) of melittin (2.4 mg/kg), also indicating no damage

Comparison of the relative weights (g/100 g body weight) of the liver (7.08 ± 0.75 vs. 7.19 ± 1.45, *P* = 0.87) and kidney (0.96 ± 0.09 vs. 0.80 ± 0.23, *P* = 0.35) showed no statistical difference between control and melittin-treated animals. In line with biochemical findings, histopathological assessment of liver and kidney tissues showed typical structure without any apparent alterations in melittin-treated mice (Fig. 2).

### *In vitro* antibacterial results

A melittin concentration range of 2-100 µg/mL was used for MIC assay. In addition, the MBC of melittin was assessed against 14 clinical isolated nosocomial pathogens. Results of MIC and MBC of melittin are displayed in Table 2. The mean values of MIC (13.71 µg/mL) and MBC (20.08 µg/mL) of melittin for XDR *A. baumannii* and MRSA were equal. The MIC and MBC values of melittin for KPC-KP were 32 µg/mL, and 50 µg/mL, respectively.

**Table 2:** Result of *in vitro* antimicrobial activity

Strains	Source	MIC (µg /mL)			MBC (µg/mL)		
***A. baumannii***		**Melittin**	**Colistin**	**Vancomycin**	**Melittin**	**Colistin**	**Vancomycin**
XDR-CI3	Blood	16	0.5	N/A	16	0.5	N/A
XDR-CI12	Lung	8	0.25	N/A	8	0.25	N/A
XDR-CI32	Blood	16	0.5	N/A	16	0.5	N/A
XDR-CI33	Blood	16	0.5	N/A	16	0.5	N/A
XDR-CI45	Lung	16	0.5	N/A	16	0.5	N/A
XDR-CI60	Lung	8	0.25	N/A	8	0.25	N/A
XDR-CI66	Blood	16	0.5	N/A	16	0.5	N/A
XDR-CI86	Blood	16	0.5	N/A	16	0.5	N/A
Mean		13.71	0.42	-	13.71	0.42	-
***S. aureus***							
MRSA-CI26	Wound	32	N/A	0.25	32	N/A	0.25
MRSA-CI6	Lung	16	N/A	0.5	16	N/A	0.5
MRSA-CI39	Lung	8	N/A	0.25	8	N/A	0.25
MRSA-CI46	Wound	16	N/A	0.25	16	N/A	0.25
MRSA-CI47	Wound	32	N/A	0.25	32	N/A	0.25
Mean		20.08	-	0.3	20.08	-	0.3
*** K. pneumonia***							
KPC-KP-CI1	Blood	32	0.5	N/A	50	0.5	N/A

MIC: minimum inhibitory concentration; MBC: minimum bactericidal concentration; CI: clinical isolate; XDR: extensively drug-resistant; MRSA: methicillin-resistant *Staphylococcus aureus*; KPC-KP: KPC-producing *Klebsiella pneumonia*; N/A: not applicable

**Fig. 3:**
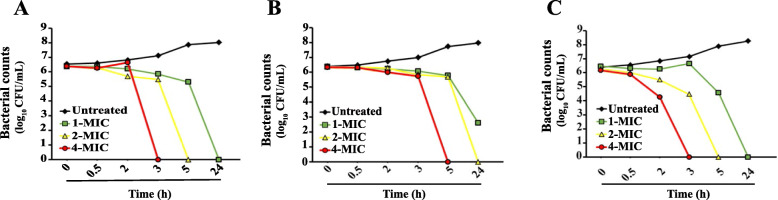
Time-kill curves performed on *A. baumannii* (**A**), S. *aureus* (**B**), and *K*. *pneumonia* (**C**) treated with 1×MIC (green line), 2×MIC (yellow line), and 4×MIC (red line) doses of melittin peptide

The TKC assay was performed to monitor cell viability versus time. The time-kill kinetic curves of melittin against XDR *A. baumannii*, MRSA, and KPC-KP were determined at 1×MIC, 2×MIC, and 4×MIC. Melittin induced a bactericidal effect in both time-dependent and concentration-dependent manners. We found that melittin killed *A. baumannii* within 3-h, 5-h, and 24-h after inoculation at 4×MIC, 2×MIC, and 1×MIC, respectively (Fig. 3 A). However, melittin exhibited a lower antimicrobial effect on MRSA, at the 1×MIC did not pose significant efficacy up to 24-h post-incubation. Melittin at 4×MIC and 2×MIC could kill the MRSA bacteria in 5-h, and 24-h post-incubation, respectively (Fig. 3B). The bactericidal efficiency of melittin against KPC-KP was similar to its effects on *A. baumannii.* Melittin at 4×MIC, 2×MIC, and 1×MIC killed the KPC-KP after 3-h, 5-h, and 24-h, respectively (Fig. 3 C).

### *In vivo* antibacterial results

#### Results of survival analysis

**Fig. 4:**
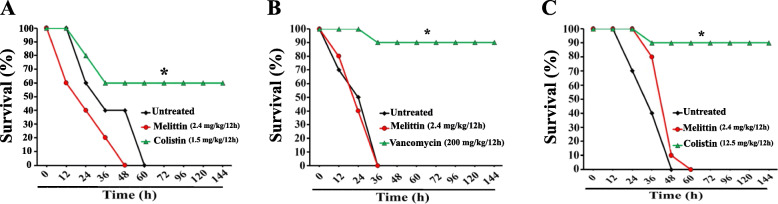
The results of the survival experiment determined the efficacy of melittin, along with untreated and colistin/vancomycin controls. The neutropenic mice (*n* = 10 each) were inoculated with a lethal amount of *A. baumannii* (A-10^7^ CFU/mouse), *S. aureus* (B-10^7^ CFU/mouse), and *K*. *pneumonia* (C-10^8^ CFU/mouse) and treated with phosphate buffered saline (PBS), melittin (2.4 mg/kg) and colistin/vancomycin as described in methods. The difference was defined as significant as *p < 0.5

Three survival experiments consisting of 30 infected mice each were used to determine the efficacy of melittin. In the first experiment, mice were infected with the LD_100_ of XDR *A. baumannii* and allocated into three groups: untreated, melittin-treated (2.4 mg/kg/12-h), and colistin treated (1.5 mg/kg/12-h). The results of survival analysis (Mantel-Cox log-rank test) revealed a statistically significant difference between groups [ χ2 (2) = 9.87, *P* = 0.007] (Fig. 4 A). The pairwise comparisons also showed that the survival rate of the colistin-treated group was statistically higher than both untreated (*P* = 0.023) and melittin-treated (*P* = 0.004) groups; meanwhile, there was no statistically difference between melittin and untreated groups (*P* = 0.42).

In the second experiment, mice were infected with the LD_100_ of MRSA and allocated into three groups: untreated, melittin treated (2.4 mg/kg/12-h), and vancomycin treated (200 mg/kg/once). The results of survival analysis (Fig. 4B) showed that there were differences in the survival distribution between the studied groups [ χ2(2) = 22.17, *P* < 0.001]. The survival rate of the vancomycin-treated group was statistically higher than both untreated (*P* < 0 0.001) and melittin treated (*P* < 0 0.001) groups; meanwhile, there was no statistical difference between melittin and untreated groups (*P* = 0.98).

The third survival experiment was performed on thirty mice infected with LD_100_ of KPC-KP. The infected animals were divided into three groups (*n* = 10 each), including untreated, melittin treated (2.4 mg/kg/12-h), and colistin treated (12.5 mg/kg/12-h.). The results of survival analysis (Fig. 4 C) showed that there was a statistically significant difference [χ2 (2) = 21.62, *p* < 0 0.001] between the studied groups. The pairwise comparisons showed that the survival rate of colistin treated group was statistically higher than both untreated (*P* < 0 0.001) and melittin treated (*P* < 0 0.001) groups; meanwhile, there was no statistical difference between melittin and untreated groups (*P* = 0.73).

#### Results of peritonitis models

To further evaluate the antimicrobial efficacy of melittin, three peritoneal models of infection consisting of 45 infected mice (each) were developed. BALB/c mice were infected by sub LD_100_ doses of XDR *A. baumannii*, MRSA, and KPC-KP. The peritoneal CFU counts and qualitative blood culture were performed at different studied time-points.

**Fig. 5:**
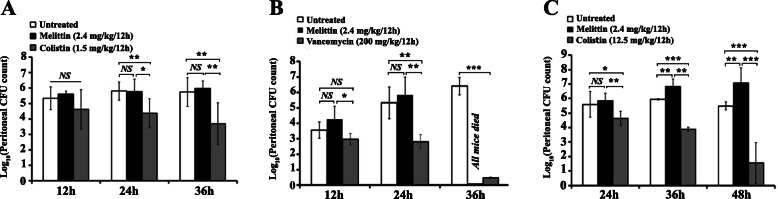
Results of peritoneal CFU assessment to determine the efficacy of melittin along with untreated and colistin/vancomycin controls. The neutropenic mice (*n* = 15 each group) were inoculated with a sub-lethal amount of *A. baumannii* (A-10^6^ CFU/mouse), *S. aureus* (B-10^6^ CFU/mouse), and *K*. *pneumonia* (C-10^7^ CFU/mouse) and treated with phosphate buffered saline (PBS), melittin (2.4 mg/kg) and colistin/vancomycin as described in methods. Results are expressed as mean ± SD. (*n* = 5 each studied time point). Difference were defined as significant as *p < 0.5, **p < 0.01, and ***p < 0.001 between groups

Melittin treatment (2.4 mg/kg/12-h) could not decrease the peritoneal loads of *A. baumannii* XDR in 12–36 h post-infection (Fig. 5 A). On the other hand, colistin (1.5 mg/kg/12-h) could statistically reduce bacterial counts in 24-h and 36-h post-infection compared with the untreated group (*P* = 0.03, *P* = 0.003 respectively). The results of blood culture also revealed that all blood samples of the untreated groups were positive up to 36-h (Table 3). In line with the findings of the peritoneal count, the results of blood culture also revealed that about 80 % of samples (4/5) of melittin treated group were positive in all studied time points. However, colistin-treated animals’ positivity of blood culture was gradually decreased from 80 % (4/5) in 12-h to 60 % (3/5) in 36-h.

**Table 3:** Results of qualitative blood culture (number of positive/ total)

Model of and Treatment		Time of blood collection			
		12 h	24 h	36 h	48 h
***A. baumannii***	**Untreated**	5/5	5/5	5/5	N/A
	**Colistin** **(1. 5 mg/kg/12 h)**	4/5	4/5	3/5	N/A
	**Melittin** **(2.4 mg/kg/12 h)**	4/5	4/5	4/5	N/A
***S. aureus***	**Untreated**	5/5	5/5	5/5	N/A
	**Vancomycin** **(200 mg/kg/once)**	4/5	0/5	0/5	N/A
	**Melittin** **(2.4 mg/kg/12 h)**	5/5	5/5	*	N/A
***K***. ***pneumonia***	**Untreated**	N/A	5/5	5/5	5/5
	**Colistin** **(12.5 mg/kg/12 h)**	N/A	5/5	3/5	3/5
	**Melittin** **(2.4 mg/kg/12 h)**	N/A	5/5	5/5	5/5

N/A: not applicable. * representing no mouse was survived in the studied time point

Melittin also showed no efficacy in MRSA peritonitis. Melittin treatment (2.4 mg/kg/12-h) did not significantly reduce MRSA loads in the peritoneal fluid than the untreated group at both 12-h and 24-h post-infection (*P* = 0.13 and *P* = 0.61, respectively). Moreover, in the melittin-treated group, the remained mice died before 36-h (Fig. 5B). Nevertheless, vancomycin treatment (200 mg/kg/once) significantly reduced peritoneal loads of MRSA in all studied time points compared to untreated groups (*P* = 0.04, *P* = 0.001, and *P* < 0.001, respectively). The results of blood culture evaluation also showed that 100 % (5/5) of blood samples of both untreated and melittin-treated groups were positive during the studied period (Table 3). Vancomycin treatment could effectively (100 %) eliminate the MRSA from blood at 24-h and 36-h post-infection.

Surprisingly, melittin treatment (2.4 mg/kg/12-h) increased peritoneal counts of KPC-KP (Fig. 5 C). Melittin significantly increased KPC-KP loads in 36-h and 48-h post-infection than the untreated group (*P* = 0.002 and *P* = 0.004, respectively). Nevertheless, then again, colistin (12.5 mg/kg/12-h) was able to reduce KPC-KP loads in all studied time points significantly (*P* = 0.03 at 24-h, *P* < 0.001 at 36-h, and *P* < 0.001 at 48-h). In line with the results of peritoneal counts, assessment of blood samples revealed that melittin treatment was unable to eliminate KPC-KP from the bloodstream (Table 3). Contrariwise, colistin treatment (12.5 mg/kg/12-h) could decrease 40 % of positive blood cultures after 48-h of infection.

## Discussion

Increasing the incidence of nosocomial infections and antimicrobial resistance threatens the effective control against bacterial infections. The ESKAPE pathogens (*Enterococcus faecium*, *Staphylococcus aureus*, *Klebsiella pneumoniae*, *Acinetobacter baumannii*, *Pseudomonas aeruginosa*, and *Enterobacter* species) are the leading causes of nosocomial infections throughout the world. A new approach is therefore needed for the development of new antimicrobial agents. Currently, colistin is an AMP that has become the only remaining alternative for treating MDR Gram-negative bacterial infections. Due to the colistin origin, AMPs have been highlighted as a promising resource for developing novel antimicrobial agents. In consequence, several AMPs have been characterized and evaluated. Melittin, the major antimicrobial component of bee venom, is one of the most studied AMPs. However, the *in vivo* data on its effectiveness and safety in systemic administration is quite limited.

In this study, we tested the safety profile of melittin using different *in vitro* and *in vivo* methods. The next step, the antimicrobial activity of melittin at its safe dose, was investigated using mouse peritoneal infection models against XDR- *A baumannii*, MRSA, and KPC-KP pathogens.

Cell viability and hemolysis assay were performed to evaluate *in vitro* toxicity of melittin. Cell viability was assessed by colorimetric MTT assay, and its result revealed that melittin displayed significant cytotoxicity against normal human fibroblast cells (IC_50_ = 6.45 µg/mL). In addition, melittin showed an extensive hemolytic activity against normal human RBCs (HD_50_ = 0.44 µg/mL). When comparing our results to previous studies, the cytotoxicity of melittin has been widely studied on cancer cell lines, while data about normal cells are limited. Similar to our findings, Duffy et al., in a recently published paper have reported that melittin exhibited cytotoxic activity with IC_50_ values ranged from 2.94 to 7.45 µg/mL in HDFa (normal primary adult human dermal fibroblasts), MCF 10 A, and MCF-12 A cell lines (human mammary immortalized epithelial cells, nontransformed). In addition, they found that IC_50_ of melittin in some breast cancer cell lines (human and murine subtypes) were ranging from 5.58 to 11.7 µg/mL. Despite this obvious overlap, they concluded that melittin was significantly more potent against breast cancer cell lines compared to normal cells [29]. Same results also reported in earlier studies where melittin treatment caused cytotoxicity in different normal and cancer cell lines with IC_50_ values ranging from 1.7 µg/mL to 5.5 µg/mL [16, 30, 31]. These findings are consistent with some previous studies indicating melittin has poor cell selectivity [13, 30]. The result of the hemolysis assay was also consistent with previous studies in which melittin showed massive hemolytic activity, indicating HD_50_ ranged from 0.5 µg/mL to 2.84 µg/mL [31–33]. Hemolysis activity is one of the most accepted signs of drug toxicity [34]. It is also the main cause of hampering the clinical development of new potential drugs such as AMPs [35]. To shed light upon the matter, we can compare the hemolysis activity of melittin with colistin as a clinically approved AMP. A recently published study showed that colistin had no hemolytic activity at concentrations up to 256 µg/mL [36]. Also the colistin IC_50_ value was reported about 113 µg/mL in RAW 264.7 cell line (macrophage) [37].

As discussed, numerous studies have shown beneficial effects of melittin on different pathogens and tumor cell lines without investigating its efficacy in normal cells and comparing it with clinically approved drugs. Although melittin exhibited high cytotoxicity against several bacteria and a broad spectrum of cancer cell lines, at the same concentrations, the peptide is also highly toxic to normal cells [14, 38, 39]. In some studies, the peptide was used as a reference lytic peptide, indicating its non-specific toxicity [35].

Since our primary aim was to evaluate the *in vivo* efficacy of melittin against nosocomial infections, the *in vivo* toxicity of melittin was performed. Accordingly, the LD_50_ of melittin was determined by injections of a two-fold concentration gradient in BALB/c mice. The LD_50_ and the maximum sub-lethal dose values for melittin were 4.96 mg/kg and 2.4 mg/kg, respectively. There are limited studies in which the LD_50_ of melittin has been investigated. A same finding (LD_50_ = 4 mg/kg) has been reported by Habermann and Zeuner [40]. In addition to systemic toxicity, Saeed et al. have reported that the intradermal LD_50_ of melittin in CD-1 Swiss albino mice was about 225 µg/mouse, equal to 7.5 mg/kg [41].

In clinical practice, single-dose therapy of antibiotics rarely captures therapeutic results, and treatment courses span days to weeks with the prescription of repeated doses of antibiotics. Therefore, we further determined cumulative toxicity by injection of 16 maximum sub-lethal doses of melittin. The results of the sub-acute toxicity assay showed that the repeated administration of melittin sub-lethal dose (2.4 mg/kg/8-h i.p.) did not show cumulative toxicity in mice. The histological examination of liver and kidney samples did not show any devastating changes in line with biochemical results. However, the hematological evaluation revealed that repeated injections of melittin (2.4 mg/kg/8-h) caused a significant decrease in WBCs count. However, the level of WBCs was remained in the normal range [42]. Similarly, Gui et al. found that a single administration of melittin up to 3.15 mg/kg did not cause renal and hepatic alterations in mice [43].

Before *in vivo* experiments, we tested the efficacy of melittin against clinical isolates of XDR- *A. baumannii*, MRSA, and KPC-KP pathogens to determine MIC, MBC, and TKC of melittin.

The MIC and MBC of melittin against XDR- *A. baumannii* were higher than colistin (8–16 µg/mL vs. 0.25–0.5 µg/mL). The results of TKC also showed that melittin induced a bactericidal effect in both time-dependent and concentration-dependent manners against *A. baumannii*. Vila-Farres et al. have reported lower MIC and MBC values than our study. They found that melittin had the MIC value at 4 µg/mL for colistin-susceptible *A. baumannii* and 2 µg/mL for colistin-resistant *A. baumannii* [44]. Similar to our findings, Giacometti and colleagues reported that melittin showed MICs ranged from 0.50 to 16 µg/mL against twenty clinical isolates of MDR-*A. baumannii* [45].

The results of *in vitro* antibacterial effect of melittin against clinical isolates of MRSA revealed that the MIC and MBC values of melittin ranged from 8 to 32 µg/mL. In Dosler and Gerceker’s study, the MIC and MBC values of melittin against MRSA have been reported as 2 and 0.5-4 µg/mL, respectively [9]. A recently published study also showed that melittin exhibited antimicrobial activity against MRSA with MIC and MBC values 6.7 µg/mL and 26.0 µg/mL, respectively. Also, they have found that 1×MIC of melittin had 3-log^3^ killing potential against MRSA in 2-h [46]. However, we found that melittin up to 4 ×MIC could not reduce MRSA bacterial count in the first 3-h. The TKC results of Dosler and Gerceker’s study were in agreement with our findings. They reported that melittin at 1×MIC could not significantly reduce the bacterial count in 2–8 h post-exposure [9].

We found that the MIC and MBC of melittin against KPC-KP were 32 µg/mL and 50 µg/mL, respectively. To our knowledge, there is no study investigating the antibacterial efficacy of melittin against KPC-KP strains. A previous study investigated the effect of melittin against *K. pneumonia* (susceptible) and reported the MIC ranges 4–64 µg/mL [47].

*In vivo* antibacterial efficacy of melittin was tested using a peritoneal mouse model of infections against three nosocomial pathogens: XDR- *A baumannii*, MRSA, and KPC-KP. The results of *in vivo* antibacterial efficacy were frustrating. Only colistin and vancomycin (approved drugs) showed a significant improvement in survival outcomes of infected animals (50–90 %). Melittin could not decrease peritoneal bacterial loads (in all models), while colistin and vancomycin significantly decreased bacterial counts in different studied time points.

To the best of our knowledge, a limited number of studies evaluated the systemic efficacy of melittin in animal models of infection. For example, Choi et al. investigated the effect of melittin (2.5-5 µg/mL) on the survival rate of MRSA-infected mice. They have found that all infected mice died following treatment with melittin at 2.5 mg/kg, but 50 % of the mice treated with 5 mg/kg of melittin were survived after 24-h of infection. However, they did not evaluate peritoneal bacterial loads in the survived mice [12].

## Conclusions

The results obtained from this study clearly showed that melittin has high levels of toxicity, and at its safe dose, could not exhibit a significant antimicrobial efficacy. Without a doubt, these results indicate that the toxic potential of melittin is overwhelming its antimicrobial benefits. Therefore, severe toxicity and poor antimicrobial activities of melittin hinder its application in clinical practice. These findings indicate that *in vitro* results are not necessarily translated to *in vivo* outcomes; consequently, both studies are necessary to understand a drug’s safety and efficacy.

## Data Availability

The datasets used and/or analyzed during the current study are available from the corresponding author on reasonable request.

## Abbreviations

i.p.: Intraperitoneal
XDR: Extensively drug-resistant
MRSA: Methicillin-resistant *Staphylococcus aureus*
KPC-KP: KPC-producing *Klebsiella pneumonia*
MDR: Multiple drug resistance
PDR: pandrug-resistant
AMPs: Antimicrobial peptides
DMEM: Dulbecco’s modified Eagle’s medium
FBS: Fetal bovine serum
PBS: Phosphate buffered saline
RBCs: Red blood cells
LD_50_: median lethal dose
AST: aspartate transaminase
ALT: alanine transaminase
EDTA: ethylene diamine tetra-acetic acid
MHB: Mueller-Hinton broth
MHA: Mueller-Hinton agar
BHIB: Brain heart infusion Broth
BA: Blood-agar
TSB: Trypticase soy broth
MIC: minimum inhibitory concentration
MBC: minimum bactericidal concentration
TKC: time-killing curve
LD_100_: minimal lethal dose
SD: Standard deviation
HD_50_: concentration causing 50 % hemolysis of red blood cells
